# Circulating Cell-Free DNA-Based Comprehensive Molecular Analysis of Biliary Tract Cancers Using Next-Generation Sequencing

**DOI:** 10.3390/cancers14010233

**Published:** 2022-01-04

**Authors:** Szilvia Lilla Csoma, Judit Bedekovics, Gergő Veres, Anita Árokszállási, Csilla András, Gábor Méhes, Attila Mokánszki

**Affiliations:** 1 Department of Pathology, Faculty of Medicine, University of Debrecen, H-4032 Debrecen, Hungary; csoma.szilvia@med.unideb.hu (S.L.C.); bedekovics.judit@med.unideb.hu (J.B.); gabor.mehes@med.unideb.hu (G.M.); 2 Division of Radiology and Imaging Science, Department of Medical Imaging, Faculty of Medicine, University of Debrecen, H-4032 Debrecen, Hungary; veres.gergo@med.unideb.hu; 3 Department of Oncology, Faculty of Medicine, University of Debrecen, H-4032 Debrecen, Hungary; arokszallasi.anita@med.unideb.hu (A.Á.); andras.csilla@med.unideb.hu (C.A.)

**Keywords:** biliary tract cancers, cholangiocarcinoma, liquid biopsy, cell-free DNA, estimated tumor volume, DNA yield, mutation profiling, next-generation sequencing (NGS)

## Abstract

**Simple Summary:**

In the era of personalized oncology, next-generation sequencing plays an important role in identifying mutations that may predict the molecular pathomechanism and manage biliary tract cancers (BTC) therapy. The peripheral blood of cancer patients represents variable amounts of cell-free DNA (cfDNA) released from the tumor. Tumor-derived cfDNA in BTCs also allows the effective monitoring of the molecular genetic profile and the response to chemotherapy. Our study aimed to identify genetic aberrations in cell-free and matched tumor DNA in BTCs. We assume that the efficacy of the LB-based sequencing provides a novel perspective for BTCs therapy.

**Abstract:**

Biliary tract cancer (BTC) is a rare malignancy with a long disease course and an overall poor prognosis. Despite multiple chemotherapy agents, there is no defined second-line treatment opportunity for advanced BTCs. In the era of precision oncology, NGS plays an important role in identifying mutations that may predict the molecular pathomechanism and manage the BTC therapy. The peripheral blood liquid biopsy (LB) of cancer patients represents variable amounts of cell-free DNA (cfDNA) released from tumor foci of any anatomical location. Our study aimed to identify somatic mutations and tumor variant burden (TVB) in cell-free and matched tumor DNA. We found a positive correlation between the estimated tumor volume and cfDNA yield (*r* = 0.9326, *p* < 0.0001). Comparing tissue and LB results, similar TVB was observed. SNVs were proven in 84% of the cases, while in two cases, only the LB sample was informative for molecular analysis. The most important aberrations in BTCs, such as *FGFR2*, *IDH1*, *IDH2*, *KRAS*, and *TP53*, could be detected in matched LB samples. Our prospective study demonstrates a minimally invasive testing approach to identify molecular genetic alterations in cholangiocarcinoma and gallbladder cancers. Clinical applications of cfDNA reflect by capturing the outstanding spatial tumor heterogeneity and guarantee novel aspects for the precision oncology treatment.

## 1. Introduction

Biliary tract cancers (BTCs) are rare malignancies with an extended disease course with an inadequate prognosis and restricted oncotherapeutic options [1,2]. The epithelial cells from three distinct anatomic locations are responsible for BTCs’ transformation, resulting in three BTC subtypes, which are as follows: intrahepatic cholangiocarcinoma (IHCC), extrahepatic cholangiocarcinoma (EHCC), and gallbladder carcinoma (GBC). Over the past few decades, the prevalence and mortality ratio of BTCs have been increasing [3]. According to a recent study, the five-year survival rates for IHCC and EHCC are 12 and 30%, respectively [3]. In clinical and histopathological views, the BTC subtypes have variable features, and they are often treated similarly [4,5]. Surgery is the curative approach; however, BTCs are often unresectable, gemcitabine chemotherapy and adjuvant with capecitabine remain the basic curative decision in BTCs treatment [6,7,8]. Gemcitabine was not effective in the PRODIGE 12 and BCAT trials [9,10], in this case, second-line treatment is currently defined as FOLFOX based on the ABC-06 landmark trial [11]. Nowadays, some studies highlight immunotherapy’s effectiveness in BTC management in cases with high expression of programmed death ligand-1 (PD-L1) and programmed death-1 (PD-1) [12,13,14].

Despite multiple chemotherapy agents, there is no accurate second-line treatment opportunity for advanced BTCs. The reason for the lack of therapeutic consensus is differences in underlying tumor etiology, and consequently, the three BTC subtypes have different molecular profiles [2,15,16,17]. The ideal solution is to find practical molecular genetic aberrations that may increase the oncological management of BTC cases. Next-generation sequencing (NGS) is a popular available technology with a wide spectrum of genes in an individual platform. In the era of personalized medicine, NGS plays an essential role in detecting and annotating aberrations that may predict the prognosis and management of BTC therapy. Despite this substantial approach, the raised difficulty of molecular factors promoting genetic variability in GBC requires a more accurate application of NGS to this malignancy [18].

The peripheral blood (PB) of cancer patients contains varying and fluctuating amounts of cell-free DNA (cfDNA) emitted from tumor foci of any anatomical location. The principle of the minimally invasive liquid biopsy (LB) has been successfully transferred to clinical routine follow-up of solid tumors [19]. Cell-free nucleic acids spread at extremely low levels in the PB, and consequently, detection of molecular genetic alterations demands high-throughput techniques, such as NGS. A small amount of literature data reflecting LB for BTC patients were published [20,21], although studying cfDNA has enormous clinical possibilities, such as in cases where it is difficult to carry out tissue biopsy (e.g., liver or gallbladder). PB sampling has minimal procedural risks, suitable for longitudinal follow-up of the patients, and with the help of LB, the effectiveness of the chemotherapy can be monitored.

The aims of our study were (i) to characterize histological and immunohistochemical (IHC) staining features of samples originating from the three subtypes of BTC patients, (ii) to quantify tumor-derived cfDNA, (iii) to find a correlation between estimated tumor volume (ETV) and nucleic acid concentration, (iv) to identify somatic mutations and tumor variant burden (TVB) in LB samples, (v) to compare alterations between histological and LB samples, and (vi) to elucidate genetic difference among the three subtypes of BTCs. For this purpose, histologic examination, IHC, ETV calculation, nucleic acid isolation from the two types of samples (tissue and LB), and NGS gene panel analysis targeting 67 genes (Archer VariantPlex Solid Tumor panel, Illumina MiSeq platform) were performed on all samples of 25 BTC patients.

## 2. Materials and Methods

### 2.1. Patients Samples

Altogether, 25 formaldehyde-fixed paraffin-embedded tissue (FFPE) and 25 paired PB LB BTC samples were tested. Because of the long disease course, LB was performed for the mean 24 months (range: 12–36) after initial tissue biopsy (IHCC and EHCC cases)/surgical resection (only GBC cases) to evaluate chemotherapy effectiveness. All protocols have been approved by the author’s respective Institutional Review Board for human subjects (IRB reference number: 60355-2/2016/EKU and 4648-6/2018/EÜIG).

### 2.2. Tumor Volume Evaluation

The tumor mass parameters have been determined by using computed tomography (CT) and/or magnetic resonance imaging (MRI) right before the initial tissue biopsy/resection. According to radiological practice, maximal tumor width (w) and length (l) were measured. Estimated tumor volume (ETV) has to be calculated using the following formula:

V (mm^3^) = w^2^ (mm^2^) × l (mm) ÷ 2

### 2.3. Histology and Immunohistochemistry

Hematoxylin and eosin (H&E) stainings were performed and carefully analyzed by pathology specialists. The samples containing at least 20% tumor cells were selected for DNA isolation. IHC of CK7 (clone OV-TL 12/30, 1:100 dilution, Dako, Agilent Technologies, Santa Clara, CA, USA), CK8/18 (clone 5D3, 1:100 dilution, Leica Biosystems, Wetzlar, Germany), CK19 (clone Ks19.1, 1:200 dilution, Biocare Medical, Pacheco, CA, USA), CK20 (clone Ks20.8, 1:200 dilution, Leica Biosystems, Wetzlar, Germany), CA19-9 (clone C241:5:1:4, 1:200 dilution, Leica Biosystems, Wetzlar, Germany), HSA (clone OCH1E5, 1:700 dilution, Dako, Agilent Technologies Company, Santa Clara, CA, USA), arginase (polyclonal, 1:20,000 dilution, Merck, Darmstadt, Germany), and glypican-3 (clone 1G12, 1:50 dilution, Biocare Medical, Pacheco, CA, USA) were performed to validate BTC diagnosis. PD-L1 IHC (clone SP142, 1:100 dilution, Abcam, Cambridge, UK) was carried out to study the possibility of immunotherapy.

### 2.4. DNA Isolation

QIAamp DNA FFPE Tissue Kit (Qiagen, Hilden, Germany) was applied for FFPE tissues genomic DNA (gDNA) extraction. The isolations were carried out according to the manufacturer’s instructions and the gDNA was eluted in 50 µL elution buffer.

EDTA anticoagulant blood samples were centrifuged at 3000× *g* for 10 min. To eliminate cell residues, 5 ± 0.1 mL plasma was spun down (16,000× *g*, 10 min). Cell-free DNA was extracted from PB plasma into 35 µL elution buffer using QIAamp Circulating Nucleic Acid Kit (Qiagen, Hilden, Germany).

The DNA concentrations were measured in the Qubit dsDNA HS Assay Kit using a Qubit 4.0 Fluorometer (Thermo Fisher Scientific, Waltham, MA, USA).

DNA yield was calculated with the following formula:

DNA yield (ng/mL plasma) = DNA concentration (ng/µL) × elution volume (35 µL)/plasma volume (mL).

### 2.5. Next-Generation Sequencing

The amplifiable DNA amounts were estimated according to the Archer PreSeq DNA Calculator Assay Protocol (Archer DX, Boulder, CO, USA). Libraries of the samples were constructed using Archer VariantPlex Solid Tumor Kit (Archer DX, Boulder, CO, USA). The final purified libraries were quantified with KAPA Universal Library Quantification Kit (Kapa Biosystems, Roche, Basel, Switzerland).

The indexed libraries were then submitted to Illumina MiSeq System (MiSeq Reagent kit v3 600 cycles, Illumina, San Diego, CA, USA). The libraries pooling, denaturation, and dilution were carried out according to the manufacturer’s instructions. The terminating loading concentration was 8 pM libraries and 1% PhiX. Captured libraries were sequenced with a paired-end run to obtain 2 × 150 bp reads with at least 250× depth of coverage.

The fastq files were analyzed with the Local Virtual Machine application of Archer DX Analysis software (version 6.2.7; Archer DX, Boulder, CO, USA) for identifying single-nucleotide variants (SNVs) as well as insertions and deletions (indels). For the alignment, the human reference genome GRCh37 (equivalent UCSC version hg19) was used. The sequence quality was evaluated and the cutoff was determined to be 3% variant allele frequency (VAF) in FFPE samples and 2% in LB samples. Massive insertion/deletion (>50 bp) and compound structural changes could not be captured by the method. The results were defined using the latest version of the Human Genome Variation Society nomenclature. Individual gene aberrations were checked in the COSMIC (Catalogue of Somatic Mutations in Cancer), ClinVar, and TCGA (The Cancer Genome Atlas) databases for clinical significance. Single nucleotide polymorphisms (SNPs) were monitored using the dbSNP database. Detected pathogen variants were cross-checked in the OncoKB database for therapeutic options.

### 2.6. Statistical Analysis

Statistical analyses were performed with GraphPad Prism 9. Differences between the ETV and DNA concentration of the three subtypes of BTC patients were analyzed using an unpaired *t*-test. When comparing the average TVB of the three BTC subtypes, a nonparametric *t*-test was used. Association between the tumor volume and cfDNA concentration was examined with Pearson correlation test (Spearman’s rho calculation). A value of *p* < 0.05 was considered to be statistically significant.

## 3. Results

### 3.1. Patients Clinicopathological Characteristics

The clinicopathological features of the 25 BTC patients are shown in Table 1. The average age was 64.7 (range: 43–80). The gender distribution was 13/12 male/female. According to the tumor localization, 15 IHCC, five EHCC, and five GBC cases were evolved. The average ETV was 265,589 mm^3^ with a broad range: 3179–1,230,187 mm^3^. In five cases, metastasis was present (case 2, 3, 7, 13, and 14), the metastatic tumor volume was added to ETV. Tissue biopsy was performed in IHCC and EHCC cases, while resection was carried out only in potential operable GBC cases, as well. The oncological treatments included gemcitabine-based chemotherapy.

### 3.2. Histological Features including Immunohistochemistry

**Figure 1:**
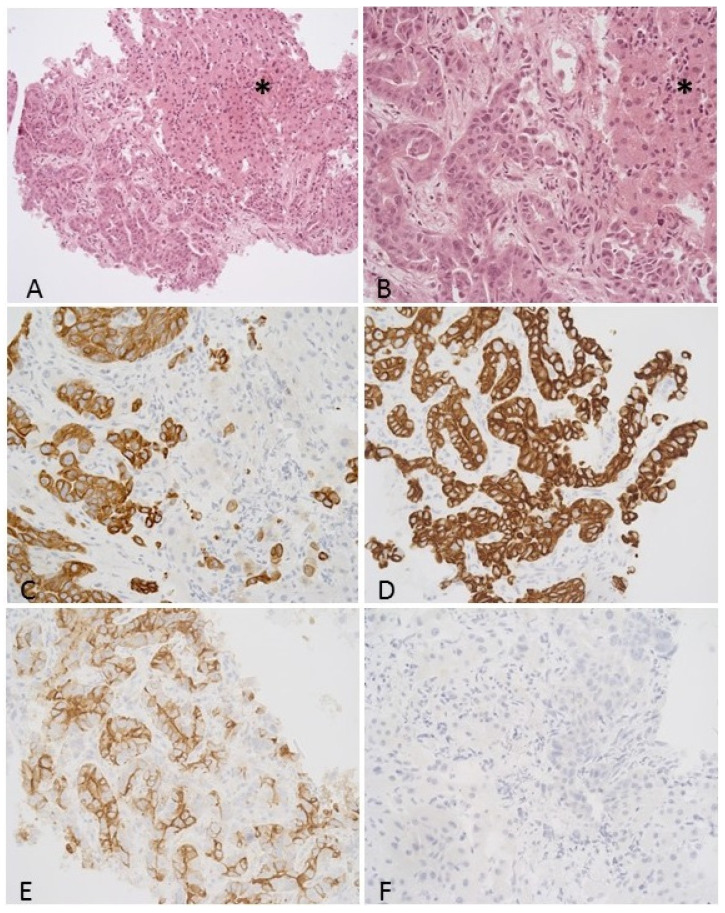
Representative histological and immunostaining of intrahepatic cholangiocarcinoma. (**A**,**B**): Core biopsy of a hepatic lesion which was consistent with intrahepatic cholangiocarcinoma (H&E, 200× and 400× magnification, respectively). The tumor is made up of irregular, atypical glandules with infiltrative growth. The neoplastic glandules are surrounded by desmoplastic stroma. Non-neoplastic hepatocytes can be seen on the right side (*). The neoplastic cells were positive for CK7 (**C**), CK19 (**D**), and Ca19-9 (**E**), while negative for CK20 (**F**) immunostaining (400× magnification).

The tumor is composed of irregularly shaped, atypical glands with infiltrative growth. Viable tumor cells without necrosis were present in all samples. Tumor cells are pleomorphic with a high nuclear/cytoplasmic ratio, marked atypia, and conspicuous nucleoli. Representative histological and immunohistochemical images are presented in Figure 1. IHC staining features of the patient samples are presented in Table 2. CK7 (24/25, 96%), CK8/18 (23/25, 92%), CK19 (25/25, 100%), and CA19.9 (23/25, 92%) positivity were found. CK20 (21/25, 84%), HSA (25/25, 100%), arginase (22/25, 88%), glypican (23/25, 92%), and PD-L1 (25/25, 100%) immunostaining negativity were performed.

### 3.3. Correlation Analysis between Estimated Tumor Volume and cfDNA Yield

No statistically significant differences were found in the ETV between the IHCC and the EHCC group (*p* = 0.9928), between the IHCC and the GBC subtypes (*p* = 0.3192), and between the EHCC and the GBC category (*p* = 0.4376), when GBC considered a different entity, as well.

The mean cfDNA yield was 35.4 ng/mL plasma (range: 6.18–98.9). No statistically significant differences were found in the cfDNA yield between the IHCC and the EHCC (*p* = 0.7736), between the IHCC and the GBC (*p* = 0.1121), and between the EHCC and the GBC entity (*p* = 0.3570).

**Figure 2:**
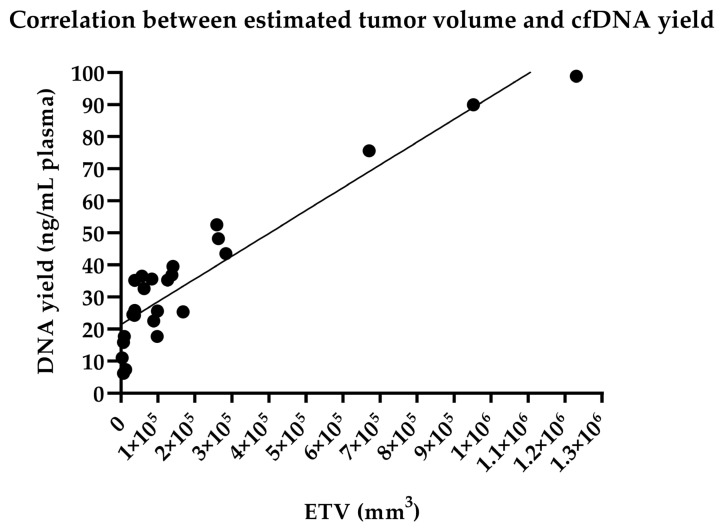
Association between estimated tumor volume (ETV) and tumor-derived circulating cell-free DNA yield. Pearson correlation coefficient was showed a significant correlation (*r* = 0.9326, *p* < 0.0001, 95% confidence interval: 0.8510 to 0.9702).

We found a positive significant correlation between the ETV and cfDNA yield using the Pearson correlation test (*r* = 0.9326, *p* < 0.0001) analyzing all BTC cases (Figure 2).

### 3.4. NGS-Based Mutation Profiling of Genomic and Cell-Free DNA

**Figure 3:**
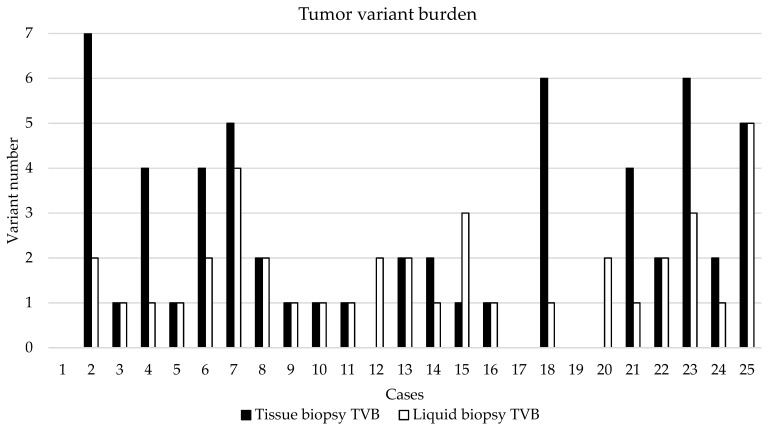
Tumor variant burden in the 25 BTC cases. TVB was defined with the number of gene variants including SNPs above 2% VAF.

TVB was defined with the number of gene variants including SNPs above 2% VAF (Figure 3). TVB was calculated in tissue and liquid biopsy samples, as well. The largest TVB was in cases 2, 18, and 23, while no nucleotide change was detected in cases 1, 17, and 19. In cases 12 and 20, only the liquid biopsy was informative for the aspect of tumor burden determination. Comparing tissue and LB results, similar TVB was observed in most of the cases.

When comparing average TVB of the three BTC subtypes, no significant difference was identified (tissue vs. LB in IHCC: 2.1 vs. 1.6, *p* = 0.3612; tissue vs. LB in EHCC: 1.4 vs. 0.8, *p* = 0.6374; tissue vs. LB in GBC: 3.8 vs. 2.4, *p* = 0.2371). No significant difference was observed, comparing tissue biopsy-derived TVB (IHCC vs. EHCC: *p* = 0.5166; IHCC vs. GBC: *p* = 0.1154, EHCC vs. GBC: *p* = 0.1281) and LB-derived TVB (IHCC vs. EHCC: *p* = 0.122; IHCC vs. GBC: *p* = 0.2034, EHCC vs. GBC: *p* = 0.0922), as well.

SNVs were detected in 21/25 patients (84%), while in cases 1, 5, 17, and 19, nucleotide aberration was not identified by our method. In two cases, only the LB sample was informative for molecular analysis (tissue biopsy insufficient for molecular analysis, case 12 and 20). Detected SNVs and their clinical significance were presented in Table 3.

The DNA VAF of tissue biopsy and LB were greatly varying with the average of 21.8% (range: 3.0–62.6) and 13.32% (range: 2.1–67.5), respectively. Pathogenic variations were proven at a rate of 17/25 (68%) and presented in some of the most usually affected genes in BTCs, such as *FGFR2*, *IDH1*, *IDH2*, *KRAS*, and *TP53*, and most of them could be identified in matched LB-originated cfDNA. Pathogenic SNVs were referred to as mutations (*n* = 28 in the 25 patients), while benign aberrations were considered neutral (*n* = 15).

**Figure 4:**
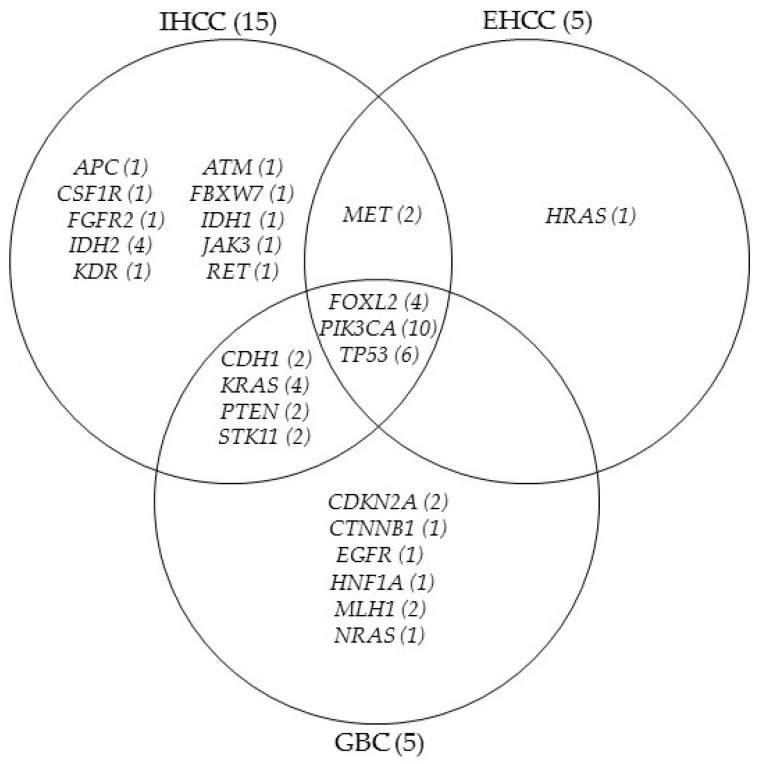
Genetically aberrant gene distribution of the three subtypes of BTCs. IHCC (15): intrahepatic cholangiocarcinoma, EHCC (5): extrahepatic cholangiocarcinoma, GBC (5): gallbladder carcinoma (case number are in bracket). Gene aberration frequencies are presented in the bracket after gene symbols.

Twenty-five genes were affected in the BTC patients. *FOXL2*, *PIK3CA*, and *TP53* SNVs have emerged in all BTC subgroups, while *CDH1*, *KRAS*, *PTEN*, and *STK11* alteration was found in the IHCC and GBC cases, as well. *MET* aberration was proved in IHCC and EHCC group, but *HRAS* c.182A>G (p.Gln61Arg) variance was detected only in one EHCC sample. Several other molecular abnormalities were identified in IHCC and GBC. The genetically aberrant gene distribution of the three subtypes of BTCs was presented in Figure 4.

## 4. Discussion

BTC is a rare malignancy with a distinctly poor prognosis and limited therapeutic options, so it would be essential to understand the molecular pathogenesis and find a specific molecular target for achieving an effective treatment [1,2]. In the area of precision oncology, not only obtaining an individual possibility of chemotherapy, but also early diagnosis is important for the oncological management of BTC patients. For this purpose, our prospective study focuses on the comprehensive genetic analysis of molecular aberrations of the background in cholangiocarcinoma.

Accessing genetic alterations, therefore, is essential for the diagnosis, management, and selection of targeted therapies, although sampling tumor tissue, when possible, is often risky and difficult to carry out. Compared to the traditional tissue biopsy, LB is not invasive and can be repeated as the potential substitute method for gaining information about the real-time molecular aberration background. The use of peripheral blood LB has been introduced in the early molecular diagnosis of several malignancies, such as lung and colorectal adenocarcinomas, and melanomas, as well [19,22,23,24]. More recently, LB application to detect aberrant gene fusions has been published for the diagnosis of aggressive lymphoma genotyping [25]. Little information was found about the utility of LB to identify diagnostic and prognostic biomarkers in BTCs [26], so for this reason we aimed to quantify and analyze cfDNA management of patients affected by BTC. No published data is available for the association between tumor mass and LB nucleic acid quantity. When determining the ETV in our study, a statistically significant correlation was proved between the tumor volume and peripheral blood plasma cfDNA concentration. The larger tumor mass, calculated based on recent in vivo CT/MR scans, was associated with higher cfDNA yield, as well. In cases with metastasis (case 2, 3, 7, 13, and 14), the metastatic tumor volume was added to ETV. LB was performed the mean 24 months after tissue biopsy (IHCC and EHCC cases), in these cases, ETV was not significantly different from the initial imaging scans. In GBC cases, where surgical resection was carried out, low cfDNA concentration was measured in the PB plasma.

Tumor Mutation Burden (TMB) is a relevant type of specific biomarker, which was introduced as a general molecular diagnostic feature to predict response to immunotherapy in a broad scale of malignancies [27]. TMB is calculated as the number of variants per Mbp genomic DNA isolated from the neoplastic tissue sample. There is very limited literature available on TMB data in association with BTCs [28]. To address this issue in our study, we calculated TVB for every case, not only for the tissue, but also in LB samples. TVB was defined in this study as the number of all gene variants including SNPs above 2% VAF. High TVB was determined to produce targeted NGS libraries of 660 regions of interest across 67 genes. Comparing tumor tissue and LB results, similar TVB was observed in most of the cases. No significant difference was identified when analyzing the average tissue biopsy-derived and LB-derived TVB of the three BTC subtypes. A total of 45 alterations were found in tissue and from these, 28 emerged in the plasma (62.2%). Controversially, four SNVs were identified only in the plasma, while in two cases, only the LB sample was informative for molecular analysis (in case 12 and 20, four variants were detected in cfDNA).

Recent reports described gene aberrations related to the individual BTC subtypes that potentially contribute to different cholangiocarcinoma pathogenesis [3,16,17,18]. In our study, SNVs were identified in twenty-one patients and the results broadly reflected the differences in the three molecular categories described in the literature [16,17,18] (Figure 4). The highest number of the affected genes (*n* = 18) was found in the IHCC type, but eight of them were shared with either EHCC or GBC types. In the EHCC group, only five gene variants could be identified, four of them were common with the other subtypes, while one (HRAS) variant occurred exclusively in this subtype. Out of the total 13 gene alterations, six were identified in GBC, which has not emerged in the other two groups. *CDH1*, *KRAS*, *PTEN*, *MET*, and *STK11* alterations were described in association with IHCC and GBC in some publications [29,30,31]. *FOXL2*, *PIK3CA*, and *TP53* variants were found in all three BTC histologic subtypes. According to the literature data, these variations are most often, but not exclusively detected in the EHCC patients, while their occurrence in IHCC samples is associated with an unfavorable prognosis [16,32,33].

One of the most common gene mutations in cholangiocarcinoma is affecting the *IDH1/IDH2* gene (encoding isocitrate dehydrogenase isotypes), which is characteristic of the IHCC category [1,3,16]. A similar finding was provided by our study, as it was detected only in IHCC patients with a frequency of 4/15 (case 2, 10, 11, and 13). In case 2, *IDH1* and *IDH2* variants emerged at the same time. *FGFR2* point mutation is one other major gene alteration in IHCC [29,30,31], which was found in a single case with pathogenic clinical significance (case 9).

Variants were further categorized according to their clinical significance as determined by the COSMIC and Clinvar databases. Pathogenic mutations were detected in 17 of 25 patients (68%). The most common affected genes in BTCs, such as *FGFR2*, *IDH1*, *IDH2*, *KRAS*, and *TP53* presented with pathogenic variants in our study population, and most of them could be identified in matched LB originated cfDNA, as well. *KRAS*, *BRAF* mutations, overexpression of *EGFR*, *HER2*, activation of oncogenic signaling pathways, DNA amplification, and deletions are associated with poor outcomes [34,35].

Detected pathogen variants were cross-checked in the OncoKB database for therapeutic possibilities. Optional oncotherapeutic agent was found in only four cases, one was specific for BTC patients. Ivosidenib for *IDH1* p.Arg132Leu aberration (case 2) was the FDA’s approved therapeutic drug for the management of advanced cholangiocarcinoma patients [36,37]. AZD4547, BGJ398, Debio1347, and Erdafitinib for all oncogenic *FGFR2* mutations, Tipifarnib for *HRAS* aberrations, and AZD8186, and GSK2636771 for *PTEN* point mutations were not specified in the FDA’s approved packaging label for BTC therapy (off-label drug).

BTCs are extraordinary rare malignancies and diagnostic sampling is frequently extremely difficult due to the special anatomical localization, partial tissue involvement, or minimal amounts of the tissue sample. Thus, our study design was also faced with such issues, which could be partially overcome when applying tumor tissue and LB-based sequencing. The major limitation of our study is the low case number originating from a single institution. The use of an NGS gene panel targeting 67 genes appeared highly effective in both sample types, although did not fully cover all affected genes described in BTCs. The presence of copy number variations (*ERBB2*) and fusions (*FGFR2*, *NTRK*) are important in BTC and are not covered in the assay, as well. LB was carried out only one time to control the chemotherapy effectiveness and no clinical follow-up was performed because most of the patients were treated with similar onco-chemotherapeutic agents.

## 5. Conclusions

Tumor-derived cfDNA in BTCs also allows the effective monitoring of the molecular genetic profile and the response to chemotherapy. The use of LB is a favorable solution because repeated invasive sampling can be avoided. We assume that the efficacy of the LB-based sequencing is increasing with the progression and cfDNA release of the tumor. In the present series, we were able to demonstrate clinically relevant SNVs from the tissue and matched LB samples of BTC patients. Our prospective study demonstrates a minimally invasive testing approach to identify molecular genetic alterations in cholangiocarcinoma and gallbladder cancers. Clinical applications of cfDNA reflect by capturing the outstanding spatial tumor heterogeneity and guarantee novel aspects for precision oncology treatment.

## Figures and Tables

**Table 1:** Clinicopathological features of the BTC patients.

Cases	Gender	Age (Years)	ETV (mm^3^)	Metastasis	BTC Subtype	Chemotherapy
1	F	80	83,349	no	IHCC	cisplatin plus gemcitabine, capecitabine, irinotecan
2	F	75	88,200	liver	IHCC	gemcitabine, bevacizumab
3	M	76	56,784	liver	IHCC	capecitabine, cisplatin plus gemcitabin
4	M	59	37,044	no	IHCC	cisplatin plus gemcitabine
5	M	68	1,680,000	no	IHCC	cisplatin plus gemcibatine, irinotecan, capecitabine
6	M	68	37,462	no	IHCC	cisplatin plus gemcitabine, capecitabine
7	M	60	259,200	peritoneum	IHCC	cisplatin plus gemcitabine, trametinib, everolimus
8	M	68	62,500	no	IHCC	cisplatin plus gemcitabine
9	F	63	1,230,187	no	IHCC	cisplatin plus gemcitabine
10	M	53	263,250	no	IHCC	cisplatin plus gemcitabine, irinotecan
11	M	70	12,800	no	IHCC	cisplatin plus gemcitabine, irinotecan, capeticabine
12	F	61	283,500	no	IHCC	cisplatin plus gemcitabine, capecitabine
13	M	75	9126	liver	IHCC	capecitabine, gemcitabine
14	F	49	140,625	ovary	IHCC	cisplatin plus gemcitabine
15	F	66	670,372	no	IHCC	gemcitabine
16	F	64	36,162	no	EHCC	cisplatin plus gemcitabine, capecitabine
17	M	43	952,544	no	EHCC	cisplatin plus gemcitabine, irinotecan
18	M	51	6664	no	EHCC	cisplatin plus gemcitabine, nivolumab
19	F	70	98,606	no	EHCC	cisplatin plus gemcitabine
20	F	71	32,000	no	EHCC	cisplatin plus gemcitabine
21	F	58	6750	no	GBC	cisplatin plus gemcitabine, irinotecan
22	M	70	126,000	no	GBC	cisplatin plus gemcitabine
23	M	68	3179	no	GBC	cisplatin plus gemcitabine
24	F	71	137,312	no	GBC	cisplatin plus gemcitabine
25	F	60	98,000	no	GBC	capecitabine, cisplatin plus gemcitabine

The number represents the case ID. Estimated tumor volume (ETV) was calculated using the following formula: V (mm^3^) = w^2^ (mm^2^) × l (mm) ÷ 2. BTC: biliary tract cancer, IHCC: intrahepatic cholangiocarcinoma, EHCC: extrahepatic cholangiocarcinoma, GBC: gallbladder carcinoma.

**Table 2:** Immunohistochemical characteristics of the patient samples.

Cases	Grading	CK7	CK8/18	CK19	CK20	CA19.9	HSA	Arginase	Glypican	PD-L1
1	G2	+	+	+	−	+	−	−	−	−
2	G2	+	+	+	+	+	−	−	−	−
3	G3	+	+	+	−	+	−	−	−	−
4	G2	+	+	+	−	+	−	−	−	−
5	G2	+	+	+	−	+	−	−	−	−
6	G2	+	−	+	−	+	−	−	−	−
7	G2	+	+	+	−	+	−	−	−	−
8	G2	+	+	+	−	+	−	−	+ *	−
9	G2	+	+	+	+	+	−	−	−	−
10	G2	+	+	+	−	+	−	−	−	−
11	G2	+	+	+	−	+	−	+	−	−
12	G3	+	+	+	+	+	−	−	−	−
13	G2	+	+	+	−	−	−	−	−	−
14	G3	+	+	+	−	+	−	−	+ *	−
15	G2	+	−	+	−	+	−	−	−	−
16	G1	+	+	+	−	+	−	−	−	−
17	G3	+	+	+	−	+	−	−	−	−
18	G2	−	+	+	−	+	−	−	−	−
19	G2	+	+	+	−	−	−	−	−	−
20	G2	+	+	+	−	+	−	−	−	−
21	G2	+	+	+	−	+	−	−	−	−
22	G2	+	+	+	+	+	−	−	−	−
23	G3	+	+	+	−	+	−	+	−	−
24	G3	+	+	+	−	+	−	+	−	−
25	G2	+	+	+	−	+	−	−	−	−

CK7: cytokeratin 7, CK8/18: cytokeratin 8/cytokeratin 18, CK19: cytokeratin 19, CK20: cytokeratin 20, CA19.9: cancer antigen 19-9, HSA: hepatocyte-specific antigen, PD-L1: programmed death-ligand 1. *: focal positivity.

**Table 3:** Detected single-nucleotide variants.

Cases	Gene	Nucleotide Change	Amino Acid Change	VAF (%) Tissue	VAF (%) LB	Clinical Significance
2	*KRAS*	c.407G>A	p.Ser136Asn	50	21.3	pathogenic
	*IDH1*	c.395G>T	p.Arg132Leu	25	19.6	pathogenic
	*PTEN*	c.925G>A	p.Ala309Thr	20	15.2	benign
	*FBXW7*	c.239C>T	p.Thr80Ile	6	0	likely pathogenic
	*IDH2*	c.332G>A	p.Gly111Asp	4.6	0	likely benign
	*CDH1*	c.596C>T	p.Thr199Ile	4.3	0	likely benign
3	*PIK3CA*	c.1634A>C	p.Glu545Ala	3	3	pathogenic
6	*FOXL2*	c.536C>G	p.Ala179Gly	57.6	15.2	benign
	*APC*	c.7504G>A	p.Gly2502Ser	49.5	6.7	likely pathogenic
	*PIK3CA*	c.1634A>C	p.Glu545Ala	3.8	0	pathogenic
7	*JAK3*	c.2164G>A	p.Val722Ile	45.5	6.9	benign
	*TP53*	c.518T>C	p.Val173Ala	13.2	4.9	pathogenic
	*KRAS*	c.35G>T	p.Gly12Val	12.8	3.6	pathogenic
8	*TP53*	c.460G>A	p.Gly154Ser	4	2.1	pathogenic
9	*FGFR2*	c.827T>G	p.Phe276Cys	37	6.3	pathogenic
10	*IDH2*	c.515G>T	p.Arg172Met	24.3	21.5	pathogenic
11	*IDH2*	c.359G>A	p.Arg120Lys	27.6	8.4	pathogenic
12	KDR	c.1444T>C	p.Cys482Arg	Low tumor cell ratio	47.2	pathogenic
	STK11	c.1189G>A	p.Ala397Thr		46.3	benign
13	*TP53*	c.536A>G	p.His179Arg	62.6	5.6	pathogenic
	*IDH2*	c.359G>A	p.Arg120Lys	30.2	4.2	pathogenic
14	*MET*	c.2975C>T	p.Thr992Ile	0	45.8	pathogenic
15	*CSF1R*	c.2916C>G	p.Cys972Trp	0	7.9	benign
	*RET*	c.1946C>T	p.Ser649Leu	0	7.8	likely pathogenic
	*FOXL2*	c.536C>G	p.Ala179Gly	61.6	4.5	benign
16	*TP53*	c.796G>T	p.Gly266Ter	29.2	5.2	pathogenic
18	*MET*	c.1124A>G	p.Asn375Ser	59.7	48.21	pathogenic
	*HRAS*	c.182A>G	p.Gln61Arg	33.3	0	pathogenic
	*PIK3CA*	c.40C>A	p.His14Asn	7.2	0	likely benign
	*PIK3CA*	c.1634A>C	p.Glu545Ala	4.6	0	pathogenic
20	*FOXL2*	c.536C>G	p.Ala179Gly	Low tumor cell ratio	67.5	benign
	*PIK3CA*	c.1571G>A	p.Arg524Lys		7.7	pathogenic
21	*NRAS*	c.104C>T	p.Thr35Ile	8.3	2.6	benign
22	*CDKN2A*	c.442G>A	p.Ala148Thr	57.8	52	benign
	*EGFR*	c.2543C>T	p.Pro848Leu	41	39	likely benign
23	*CDKN2A*	c.442G>A	p.Ala148Thr	49	52.8	benign
	*CDH1*	c.2474C>T	p.Pro825Leu	46	50.7	pathogenic
	*HNF1A*	c.862delG	p.Pro291GlnfsTer51	5.2	5.14	likely pathogenic
	*TP53*	c.325T>G	p.Phe109Val	4.9	0	pathogenic
	*PTEN*	c.802G>T	p.Asp268Tyr	4.6	0	pathogenic
24	STK11	c.842del	p.Pro281ArgfsTer6	6.3	0	pathogenic
25	*MLH1*	c.1321G>A	p.Ala441Thr	46.2	49.1	pathogenic
	*TP53*	c.707_711del	p.Tyr236LeufsTer2	12.1	3.4	likely pathogenic
	*PIK3CA*	c.1571G>A	p.Arg524Lys	11.3	5.3	pathogenic
	*CTNNB1*	c.133T>C	p.Ser45Pro	11.2	2.4	pathogenic
	*KRAS*	c.34G>T	p.Gly12Cys	6.6	0	pathogenic
	*FOXL2*	c.743T>C	p.Leu248Pro	0	3.1	likely pathogenic

NGS on samples originating from tissue biopsy and matched liquid biopsy was performed.

## Data Availability

The data presented in this study are available on request from the corresponding author. The data are not publicly available to protect the rights of patients.
